# Simple but powerful interactive data analysis in R with R/LinkedCharts

**DOI:** 10.1186/s13059-024-03164-3

**Published:** 2024-02-05

**Authors:** Svetlana Ovchinnikova, Simon Anders

**Affiliations:** Center for Molecular Biology and BioQuant Center of the University of Heidelberg, Heidelberg, Germany

## Abstract

In research involving data-rich assays, exploratory data analysis is a crucial step. Typically, this involves jumping back and forth between visualizations that provide overview of the whole data and others that dive into details. For example, it might be helpful to have one chart showing a summary statistic for all samples, while a second chart provides details for points selected in the first chart. We present R/LinkedCharts, a framework that renders this task radically simple, requiring very few lines of code to obtain complex and general visualization, which later can be polished to provide interactive data access of publication quality.

**Supplementary Information:**

The online version contains supplementary material available at 10.1186/s13059-024-03164-3.

## Background

Effective data visualization has been crucial for scientific success since the first quantitative experiments. Yet, the amount and complexity of the available data have continuously grown over the last decades, while there are certain limits to how much information one can learn from a static image [1]. Excessive details and multiple overlapping layers make it harder to grasp the crux of a plot. One solution to the problem is to come up with more and more creative and elaborate types of plots, the other is to add an extra dimension by employing interactivity. The first attempts of the latter started in the 1970s [2, 3], and by now interactivity is applied ubiquitously, not only in science but also in marketing, journalism, and any other field, where there is a need to communicate data-based knowledge to an audience.

Interactive figures are engaging. They allow users to observe data from multiple self-chosen angles making the conveyed message more credible. They also bolster data exploration sparing researchers the necessity of handpicking presumably important parts of data in advance. Therefore, we believe that further integration of interactive tools in a researcher’s routine can significantly improve the quality of research.

Numerous tools [4] now provide means of interactive inspection for many specific types of data. Examples from biology include metabolic maps [5], genome assemblies [6], scRNA-Seq or other kinds of omics data [7, 8], QTL data [9], and many more. While such solutions are each tailored for one very specific type of data, there are also a number of general low-level frameworks to create interactive apps, such as D3 [10] and Vega-Lite [11], and more high-level but still general-purpose packages, such as Vega [12], Shiny, BPG [13], plotly, Bokeh, and Observable Plot.

A crucial part that is used in many of the special-purpose solutions is “linking” of charts: the user’s click on, e.g., a data point in one overview chart (the overview chart) causes details to this very data item to be shown in another chart (the details chart) [14]. Even though such linking is often what makes interactive tools for specialized purposes useful, functionality for linking in general is missing from most general-purpose frameworks. For technical reasons, such functionality is, if at all, only offered by frameworks for web programming in JavaScript―which is most unsatisfactory for bioinformatics, a field where most work is done using R and Python.

In this paper, we present R/LinkedCharts, an R package that makes it very simple to produce linked interactive plots by providing convinient R wrappers around a core built using D3. We will first review the concept of chart linking and explain why it is of so much value especially for bioinformatics data analysis and then demonstrate the versatility of our framework and justify our design decisions. We end with a Discussion on what sets our approach apart from earlier work.

## Results and discussion

### Linking charts

As its name suggests, the central concept of LinkedCharts is linking and focusing [14]: one can connect two or more plots thus that interacting with one of them affects what is displayed in the others. We illustrate the concept of linking charts with a simple example based on data from Conway et al. [15].

**Fig. 1:**
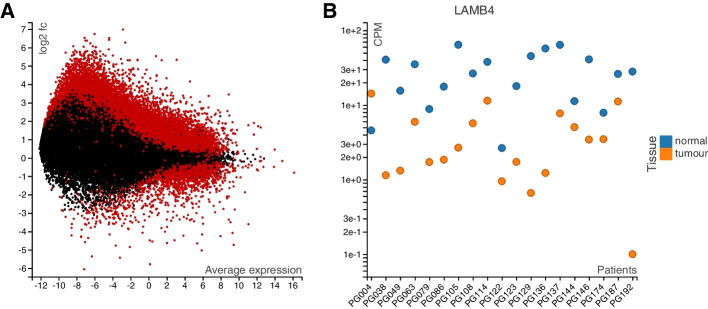
An example for two linked charts, based on a study by Conway et al. [15] comparing cancerous and normal tissues from 19 patients. The MA plot (**A**) shows all genes with their average expression on the *X*-axis and log$_2$-fold change between tumor and normal on the *Y*-axis. Red indicates genes where the difference was reported as significant by the “voom” method [18]. The plot to the right (**B**) shows, for one selected gene (here, LAMB4), the individual expression values (as counts per million, CPM) for each sample. This figure is a screenshot of a LinkedCharts app, the live version of which is provided in the supplement (as Interactive Supplementary Fig. 1): When the user clicks on any point in the MA plot (**A**), the expression plot (**B**) changes, showing the selected gene. Thus, one can rapidly gain an impression of the details hidden in a summarizing plot like the MA plot

In that study, three samples were taken from each of 17 patients with oral cancer: of normal, cancerous, and dysplasic tissue. mRNA from all these samples was sequenced to obtain gene expression values. The goal was to find genes that are differentially expressed between the tissue types―a standard task in bioinformatics, readily addressed using available software tools [16, 17]. Here, we have used the function voom from the “limma” package [18] to compare normal and cancerous tissues. It is common to visualize such a comparison with an MA plot [19], where each dot represents a gene, showing the gene’s average expression on the *X*-axis and log fold change between the two groups on the *Y*-axis (Fig. 1A). Red dots correspond to genes that are considered significantly different between the two conditions (adjusted *p*-value < 0.1).

About these genes, one may now wonder: how does the difference in expression look like for every single patient? Is it consistent across all the patients or only detected in some of them? Are there any artifacts or outliers that cause the *p*-value to be too small?

To investigate such questions, we can add another plot that shows expression values (as “counts per million”, CPM) from each individual sample (Fig. 1B). While this second plot can show expression for only one selected gene at a time, the *linking* between the two charts overcomes this limitation: in our implementation, a mouse click on a point in the MA plot causes the plot to the right to switch to displaying the expression values for the thus selected gene. Figure 1 depicts our LinkedCharts app, while a live version of the app is provided in this paper’s online supplement (https://anders-biostat.github.io/lc-paper/; also available on the Journal’s web site as Additional file 1 to the publication)―and we encourage the reader to pause for a moment and try it out there.

Of course, there are already several tools available for exploring the data from differential-expression assays (e.g., iSee [8]), and these may or may not fit the needs of a specific analysis. With R/LinkedCharts, we offer the building blocks to build such an app with minimal effort “from scratch,” while giving the analyst the flexibility to generate arbitrary plots and arbitrary linkages.

**Fig. 2:**
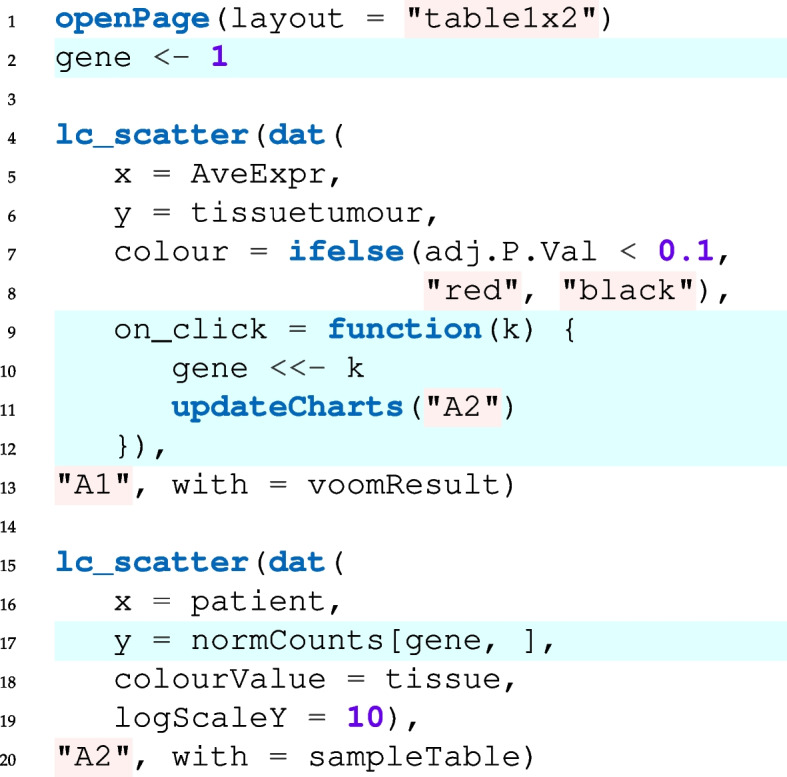
Code for generating Fig. 1. See text for details

In fact, the minimal code to set up this app takes only the few lines shown in Fig. 2 (the full code, i.e., including the code for loading the data and adjusting point colours, sizes and labels is provided in the paper supplement; code for running limma/voom, is given in our online tutorial at https://anders-biostat.github.io/linked-charts/rlc/tutorials/oscc/data_prep.html).

The two lc_scatter calls set up the two scatter charts shown in Fig. 1. In the left-hand chart (“A1”), each point depicts a gene, and *x* and *y* coordinate and point color are taken from the indicated columns of voomResult, the results table provided by the limma/voom differential-expression tool. Similarly, the right-hand chart (“A2”) takes its data from the sample table, and the *y* axis from the matrix of normalizxed read counts (that was also used as input to limma/voom).

The lines highlighted in blue cause the linking: In line 2, we introduce a global variable, gene, which stores the index of the gene to be shown in the right-hand plot. This index tells the chart which line of the normCounts matrix (where the normalized counts are stored) to use as *y* values of the expression plot (line 17). Almost every chart type in R/LinkedCharts has the on_click argument, which allows the user to define a function that is called whenever someone clicks on an element of the plot (point, line, cell of a heatmap, etc.) and is passed the index of the clicked element (k). Here, our callback function simply changes the value of gene to the clicked point index (line 10). Then, we tell R/LinkedCharts to update the second plot (line 11; “A2” is its ID set in line 20). Updating means that the package will reevaluate all arguments inside the dat() function and redraw the chart accordingly. In our case, a new value of gene will yield new *y* values for the expression plot.

This simple logic is not limited to just two plots but provides a basis to create many simple and complex apps. In the following, we will showcase a few more examples. The paper’s online supplement contains live versions for all these apps, as well as the full code to generate the apps and links to necessary data files, allowing the reader to immediately get the app in their R session and experiment with it. For all examples, we provide two versions of the code: minimal with only essential parameters needed to make the app functional and more extended with custom colors, labels, etc. In the paper, we only focus on the minimal code.

Even more example can be found in our online tutorial at https://anders-biostat.github.io/linked-charts/rlc/tutorials, including examples dealing with exploration of single-cell sequencing data.

### Event handling in R

In the simple example just discussed, the ability to link the two charts is what made the app useful and what sets R/LinkedCharts apart from other solutions for R, such as Shiny. A short technical detour might therefore be in order to explain why linking is non-trivial. Here, we first have to clarify that virtually all interactive visualization frameworks leverage the power of browser engines for HTML5 and JavaScript: the actual app is displayed by a browser. Therefore, a use interaction, such as a mouse click, is handled by the browser, and any custom event handler has to be specified using the language that the browser understands, i.e., JavaScript.

If the event handler should be written in R (such as our on_click function), the framework must provide specific functionality to connect that R code with the JavaScript code running in the browser in a manner that preserves all details of the user-interaction event. The difficulty in doing so explains why, so far, only native-JavaScript frameworks like D3 and Observable Plot, offer linking, while R- and Python-based frameworks (Shiny, Plotly, Bokeh) are (despite recent progress) still very limited with respect to offering custom event handling without having to revert to JavaScript.

The possibility to write custom event handler in JavaScript is insufficient if the user interaction should trigger a complex calculation that the analyst has already coded in their usual language of choice, here presumably in R. This is the gap that R/LinkedChart fills.

For details on how R/LinkedCharts makes it now possible to write event handlers in native R, see the “Methods” section.

### Basic syntax, chart types, and HTML5 integration

**Fig. 3:**
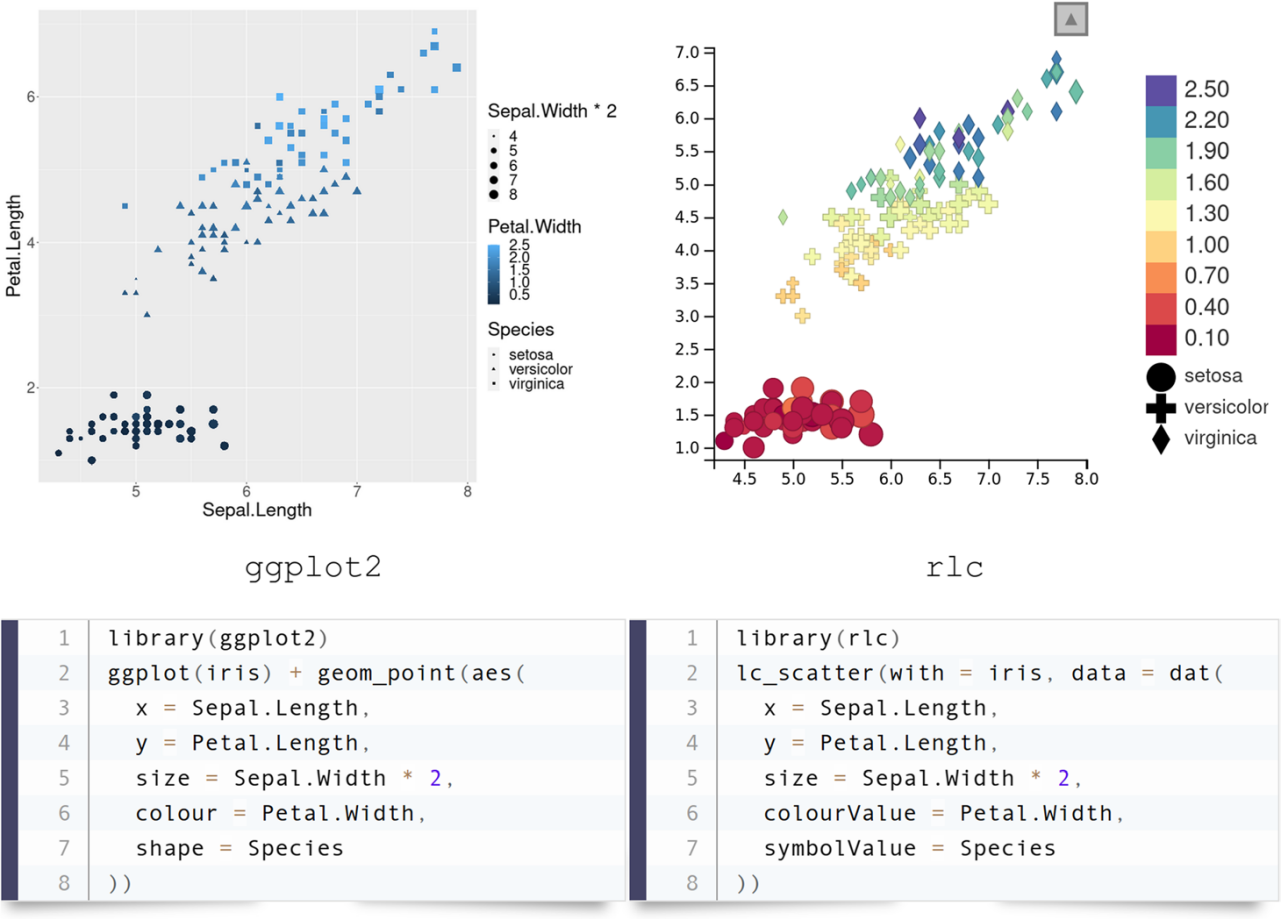
Typical syntax of an R/LinkedCharts plot with comparison to the “ggplot2” [20] package, one of the most widely used plotting libraries. Lines of the code are arranged to put the same aspects of the charts next to each other. The “iris” dataset, one of the built-in example datasets of R, was used here. Both pieces of code are complete and fully functional, and their output is shown above the code

We aimed to make R/LinkedCharts simple and familiar to any user with at least some basic knowledge of R. Every chart has a set of properties to define each of its specific aspects. In the previous example, we set the properties x, y and color, which received vectors of coordinates and colors to specify the scatter plots’ data points. This principle will be familiar to most users from other plotting libraries. For example, Fig. 3 shows a comparison of the syntax in R/LinkedCharts (“rlc” package) and ggplot (from the widely used “ggplot2” Wickham [20]) for a simple scatter plot. Lines are arranged to match the same aspects of the plots; above each code block, its output is shown. One can see that the input data structure is identical, and there is hardly any difference between the two.

**Fig. 4:**
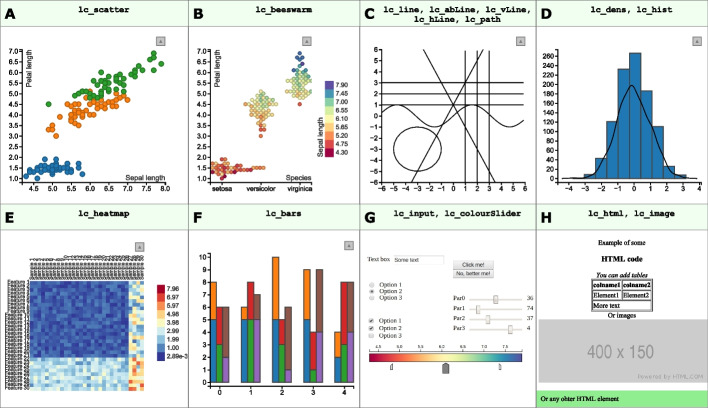
Gallery of all available plotting functions in the “rlc” package. A scatter plot (**A**); a bee swarm plot (based on the d3-beeswarm plugin of Lebeau [21]) (**B**); a collection of various lines (**C**); a histogram and a density plot (**D**); a heatmap (**E**); a bar chart (**F**); a collection of interactive elements to gather input from the user (**G**); functions to add custom HTML code and static plots to the page (**H**). All these examples with code to create them can be found in the supplement

R/LinkedCharts is not limited to scatter plots. There are 15 main functions in the “rlc” package, each generating a specific type of plot (such as scatter plot, heatmap, bar plot, etc.) or a navigation element (such as sliders or text fields). Figure 4 shows them all. Each plot is defined by its properties: some of them are required (such as x and y for a scatter plot or value for a heatmap); others are optional (palette, title, ticks etc.). A full list of all the properties with live examples is available at https://anders-biostat.github.io/linked-charts/rlc/tutorials/props.html and also on the R man page of each plotting function. For each chart type, event handlers (such as the on_click function already mentioned, and others) can be defined.

LinkedCharts apps are displayed as HTML pages, using a standard Web browser. This means that the layout, as well as decorations (such as headlines), can easily be specified by producing a standard HTML5 page, in which the elements where the charts are to be placed are marked by their id attribute. As knowledge of HTML5 is wide-spread, this allows practitioners to improve the appearance of the LinkedCharts app without having to learn anything new.

Furthermore, it facilitates integrating LinkedCharts with other web-based apps. For example, one can easily link a LinkedChars app with a web-browser-based genome browser, such as *IGV.js* [22], so that the user’s interaction with the LinkedCharts app controls what genomic region is displayed in IGV’s genome track (an example is given on the tutorial web page).

Once one has developed a rough prototype of a LinkedCharts app, the app’s appearance can be easily improved by using HTML5 to specify layout, decorations, and add further static elements. To facilitate this, the web server integrated in R/LinkedCharts provides basic functionality to also serve, e.g., images and CSS style sheets.

### Use cases

#### Interactivity for EDA and for presentation

In general, the use of interactive data visualization falls into two areas, exploratory data analysis (EDA) and data presentation and dissemination. The latter case is becoming well established: more and more authors now accompany their papers with an interactive resource to present their data and results (for example [25–27]) and allow the reader to browse through them. Typically, this chiefly serves to present and communicate research that has already been completed, and, often, it is only after most of the work on a project has been finished and the paper is being written up that researchers spend a couple of days implementing a nice-looking interactive app to accompany their publication.

However, interactive visualization has possibly even more potential in the early stages of an analysis where the analyst tries to explore new data and to get a feel for it. The reason this is so rarely done (the “interactive visualization gap” in the words of Batch and Elmqvist [28]) might be that setting up interactive visualization usually seems time-consuming and cumbersome. This is why R/LinkedCharts is designed to make it easy to rapidly create a simple app with only a few lines of code. The analyst might produce many such “quick-and-dirty” visualizations and only keep a few to later turn them into more polished works for presentation.

In the following, we will discuss use cases along this axis from early EDA to polished presentation.

#### Back-tracking in analysis pipelines

Most analysis of big data comprises multiple steps of data summarization, each reducing the total amount of data and thus losing information.

For example, in the oral-cancer example, we first have for each gene expression values from 28 samples, but the differential expression data analysis summarizes this to just 3 values: the gene’s average expression over all samples, the fold change between tumor and healthy, and the associated *p*-value. The LinkedCharts app shown in Fig. 1 allows to “undo” this summarization by inspecting the original values for each gene.

**Fig. 5:**
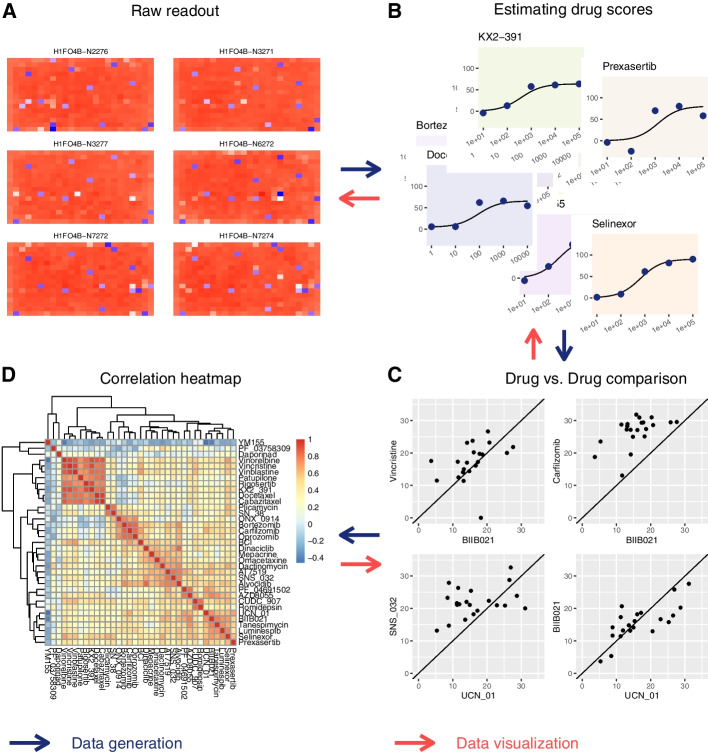
LinkedCharts can be used to “walk backwards” through an analysis pipeline. This is illustrated here using a drug screening experiment [23, 24] as an example. For an interactive version, see Interactive Supplementary Fig. 4. The *blue arrows* show the direction of a typical analysis pipeline used in drug screening experiments. We start with reading intensity values from plates with different cell lines cultured in the presence of studied drugs (**A**). These values are then normalized and turned into a fraction of the cells that remained viable. A sigmoid curve is fitted to the obtained viability values at different drug concentrations, and the area under the fitted curve yields a single score for each drug (**B**). Different drugs’ scores are compared to each other across all the tested cell lines (**C**). A drug-drug correlation heatmap is then produced to identify clusters of similar drugs (**D**). The *red arrows* illustrate the direction of interactive data exploration: We start by showing the summary heatmap plot (**D**). Suppose the researcher is interested in a particular drug combination or a cluster of drugs. In that case, he or she can examine the corresponding drug scores simply by clicking on the heatmap cell (**D**) to see the underlying correlation plots (**C**). Similarly, one can click in a point in (**C**) to examine the individual viability values at the tested concentrations and check the sigmoid fit (**B**). And finally, if needed, it is possible to take one more step back and to look at the raw read-outs to inspect them for the presence of any artifacts (**A**)

As an example of an analysis pipeline with multiple data-reduction steps, we use the drug-screening study of Ozkan-Dagliyan et al. [24]. A collection of drugs was tested against various pancreatic cancer cell lines at several concentrations per drug. Figure 5 illustrates a possible analysis pipeline: Panel A shows the viability read-out from the microtiter plates. For each combination of one cell line and one drug, the values for the different tested concentrations can be shown as a scatter plot, with each point depicting the viability value from one well (panel B). Here, we can fit dose-response curves, which can then be further summarized to a single number, such as the area under the curve, or, in the case of this study, a refined variant of that, called the drug sensitivity score (DSS) [29]. If two drugs show effect on the same subset of cell lines, they likely have similar modes of action. Hence, to assess the similarity for each pair of drugs, we compare their activity over all cell lines, as shown by the scatter plots in panel D, where each point represents a cell line, with its *x* and *y* coordinates denoting the drug sensitivity scores of that cell line for the two compared drugs. Again, we summarize each such plot into a single number, the correlation coefficient, and finally, we depict all the correlation coefficients in a correlation-matrix heatmap (panel C).

Often, such an analysis pipeline is fully automated and no one ever looks at the intermediate plots. Inspecting them is, however, crucial to note problems with data quality or mistakes in the design or programming of the analysis pipeline.

LinkedCharts allows to “walk” such an analysis pipeline backwards: in the supplement, we show an app that depicts the plots of Fig. 5 in an interactive fashion, as follows. As each cell of the final heatmap (panel C of Fig. 5) summarizes on a scatter plot comparing two drugs (panel D), we can click on any cell in the heatmap and then see the corresponding scatter plot. Each point in that scatter plot represents a pair of drug sensitivity scores, which are, themselves, summaries of dose-response curves. Again, clicking on a point in the correlation scatter plot will display these two dose-response curves. Finally, each value in a drug response curve stems from a well in a microtiter plate, and hovering over a point there hence highlights the well in a heatmap depicting the plate.

Thus, LinkedCharts allows to explore the “parentage” of any result value. If we find a specific drug-drug correlation value suspicious or surprising, or if we just wish to double-check it before drawing further conclusions from it, we can check its provenance in arbitrary detail. Similarly, we can perform random spot checks.

Each layer in the backwards journey can inform about another type of problem: from the correlation scatter plots, we may find that the correlation coefficient was unduly influenced by a single out-lying cell line; from the dose-response plot, we may find that specific dose-response curves fail to have the expected sigmoid shape; and from inspecting plate plots, we may trace back a surprising final result to, say, a normalization issue or a plate-edge effect.

Once such an analysis pipeline has been developed, all the intermediate results are typically available in suitable data structures, which can be readily explored with LinkedCharts. The supplement provides code for the example just described.

#### Quality assurance thresholds

Typically, analysis pipelines include steps to exclude bad-quality data. Often, this is done by calculating quality metrics and setting thresholds. In the drug screen example, the goodness of fit of the dose-response curves might be quantified by the residual sum of squares, and if this value exceeds a threshold, the drug sensitivity score might be discarded as unreliable. In the oral-cancer sample, the log fold change of some genes might be unduly influenced by a single outlying sample, and one might use a threshold on an outlier-detection score such as Cook’s distance to flag such genes.

Typically, the thresholds on such quality metrics are chosen a priori, often simply taking over values from previous work or from tutorials, even though the characteristics of the assay might have changed. Doing otherwise seems to cause a chicken-and-egg problem: one cannot run the analysis without first somehow deciding on thresholds, and therefore, one cannot use analysis results to guide the choice of thresholds.

The approach of “walking the pipeline backwards” with LinkedCharts opens another approach: typically, outliers tend to cause false positive results. Therefore, one can run the analysis first without excluding any outliers, then inspect the provenance of the statistically significant items found and will be so guided to specifically those places in the raw data where outliers can actually cause false positives. This provides the analyst with a better “feel” for the data and the analysis procedure and helps build an intuition that will allow to more critically judge whether traditionally used standard values for quality-assurance thresholds are appropriate for the specific data set under analysis.

#### Exploratory analysis

Analyzing complex data sets from many different angles and asking many different questions about them is crucial to all computational biology, not only to ensure that one does not overlook potential problems but also in order to not miss the chance of serendipitous discoveries. The importance of such exploratory data analysis (EDA) has been argued since long, and it therefore forms a large part of computational biologists’ everyday work. An important element is to pick examples and study them in detail, similarly to the quality-assurance applications discussed in the previous section, but now with the aim of getting a “feel” for the data and looking for insights.

**Fig. 6:**
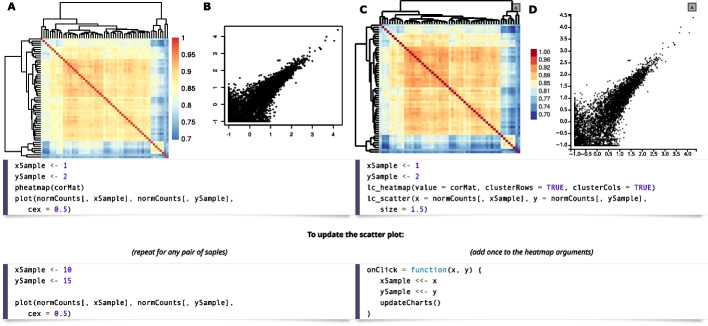
An example of an R/LinkedCharts app (**C**, **D**) for a simple exploratory analysis and the code to generate it in comparison with static plots (**A**, **B**) produced for the same purpose. The heatmaps (**A**, **C**) show Spearman correlation of gene expression for all samples from Conway et al. [15]. Here, we can see, inter alia, two outlier samples in the heatmap’s bottom-right corner and some more or less pronounced clusters of samples with similar gene expression levels. The scatter plots (**B**, **D**) show expression values for two samples plotted against each other. Browsing through several such plots can help the researcher get a feeling of the data and explore unexpected patterns like the outliers just mentioned. The code is split into two pieces, where the upper one is responsible for generating the plots and the lower part shows the code to update them to show a specific sample pair. For static plots, one has to execute the same lines of code for any pair of samples, while for R/LinkedCharts the provided code should be added to the list of arguments for the heatmap. After that, switching between pairs of samples can be done simply by clicking on the corresponding cell of the heatmap. The static heatmap (**A**) was generated with the “pheatmap” package [30]; scatter plot (**B**) was made with a base R function. The live version of the app can be found in the supplement. For simplicity, gene expression for all the samples is subset to 8000 randomly selected genes

The standard approach in inspecting examples is to pick, e.g., a gene from a result list, produce a plot showing the provenance of this result, then pick another gene, change the code for plotting to now show underlying data for this gene, etc. At that point, it is trivial to alter this code into a linked charts app, using the similarity between code for static and dynamic plots (Fig. 6).

Figure 6 illustrates this with another example based on the oral cancer dataset. A bioinformatician had produced a correlation heatmap depicting correlations between all sample pairs (Fig. 6A), using, e.g., the pheatmap package [30], and now wishes to inspect a specific correlation value (panel B) and writes to this end the short code shown in the figure. To inspect other sample pairs, she would simply change the sample indices in the code. This is routine practice for most bioinformaticians, but cumbersome. As the code example below the plots in Fig. 6 shows, however, it is now virtually none effort to transform the code into a LinkedCharts app, by merely making a few simple substitutions.

#### Public apps and concurrent use

Technically, an R/LinkedCharts app is provided by a web server running inside the R session and can hence be used from any web browser. Importantly, there is no need for that web browser to be running on the same computer as the R session. This allows a bioinformatician to easily share a LinkedCharts application with colleagues. They only have to direct their operating system’s firewall to open the TCP/IP port the app is listening at for incoming connections and tell their colleagues their computer’s IP address or DNS name and the port number, which they simply enter into their browser’s address line.

As now multiple users might use the app simultaneously, we have to make sure that each user gets their own copy of any global variable, such as the variable gene in the initial code example. To do so, a trivial change is required: one only has to list all such session variables at the beginning. In the initial code example, one would simply amend the first line to 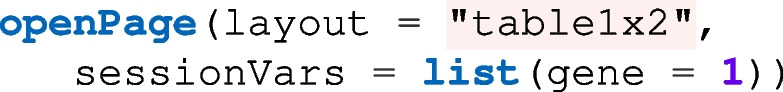


#### Apps with complex user interfaces

**Fig. 7:**
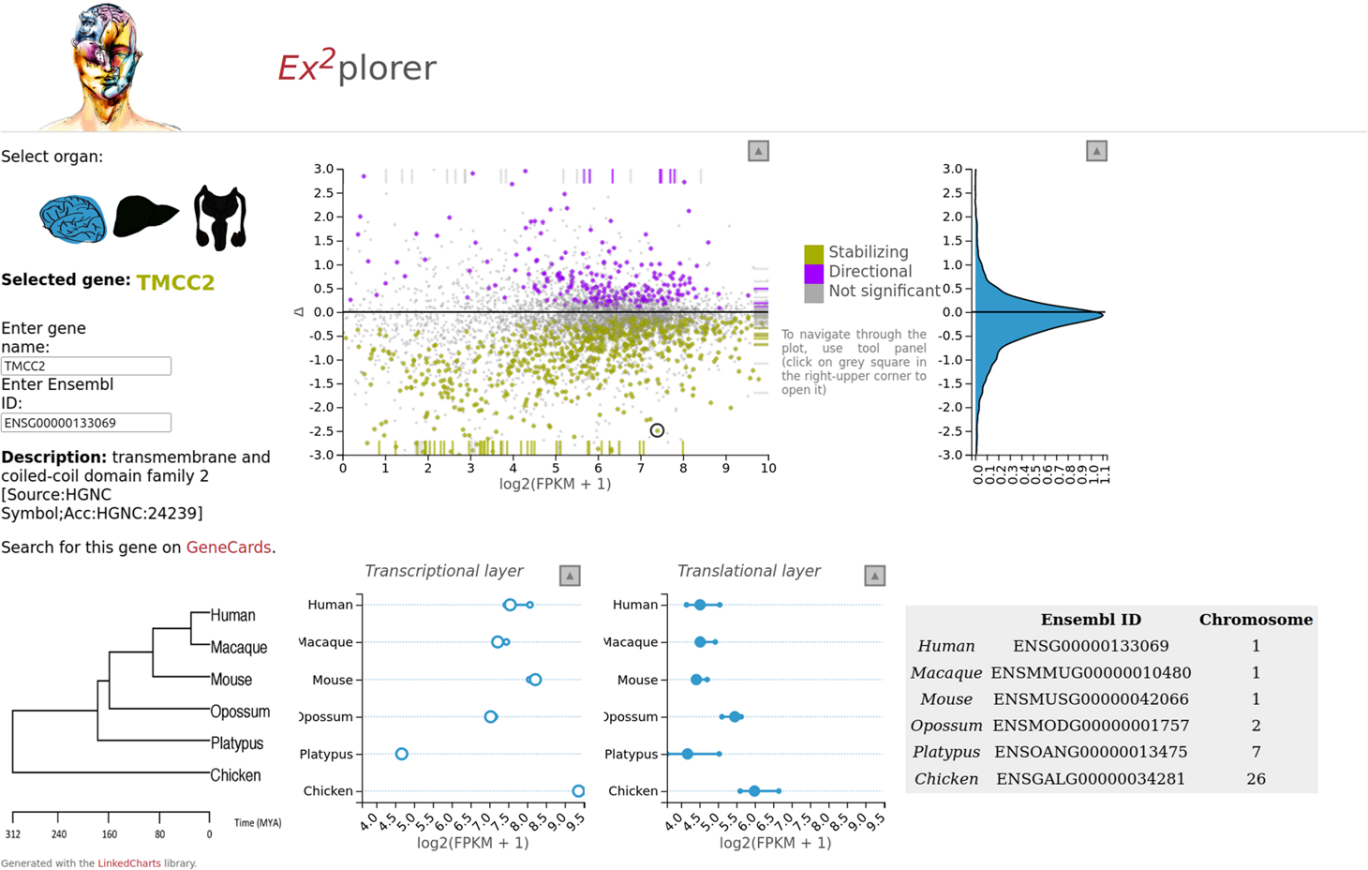
A screenshot of a paper supplement [31] made with LinkedCharts. The main chart (upper row, center) shows for every gene in the study its average expression and so-called $\Delta$-score, which indicates whether evolutionary changes in the translatome compensate for changes in the transcriptome or introduces additional variance. The two plots below show expression values for the selected gene in all the tested samples. The user selects a gene by clicking on the corresponding point of the main plot or by entering the gene name (upper-left corner). The density plot to the right shows the distribution of $\Delta$-scores, and its *Y*-axis is linked to the *Y*-axis of the main plot. In the upper-right and bottom-left corners, some additional information on the selected gene is displayed. Icons in the upper left corner allow switching between the three studied tissues. Detailed information on the data, study goal and the source code for the app can be found in the related publication. The app is written in JavaScript and, thus, can be downloaded and opened in any modern browser without installation requirements. Though largely customized, the app is based on the same principles as other examples throughout this paper. For the live version, see https://ex2plorer.kaessmannlab.org/

In all examples discussed so far, user input is constrained to selecting data points in one chart in order to affect the display in a linked chart. However, the LinkedCharts library also provides for more general means of data input by the user, by leveraging the HTML5 tag <input> and thus offering buttons, checkboxes, radio buttons, scrolls, and text fields via the “rlc” function lc_input (Fig. 4G). As for any other LinkedCharts element, lc_input can be provided a callback function that is run every time the user changes the state of an input element (e.g., clicks a button or enters new text). This allows to easily add functionality to enter, say, a gene name rather than clicking on its point (as in Fig. 7) but also to build up complex apps.

**Fig. 8:**
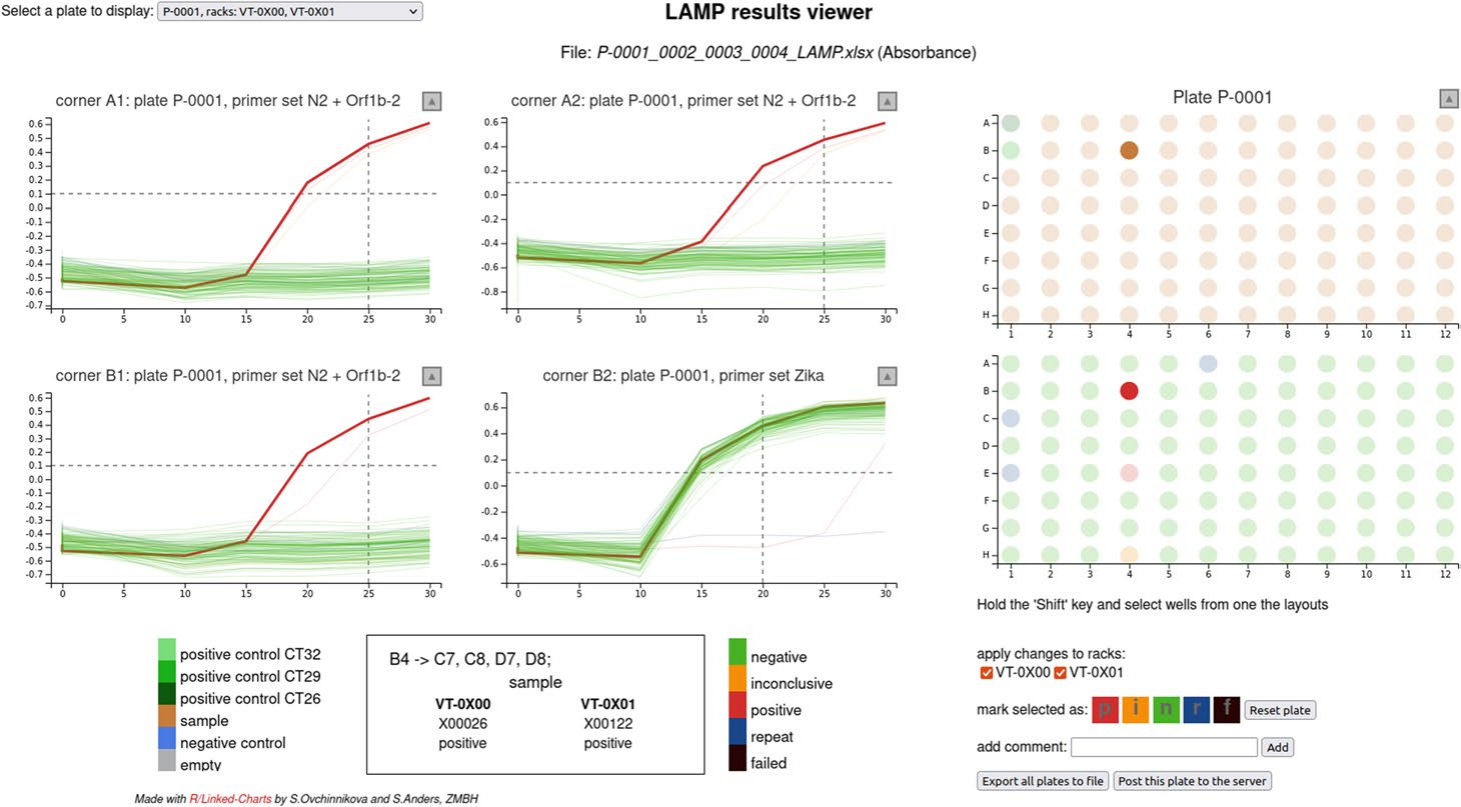
A screenshot of an app that was used as a GUI to perform manual inspection and classification of LAMP testing for SARS-CoV-2 viral RNA [32, 33]. The app was used during our SARS-CoV-2 surveillance study [34] and for voluntary testing for COVID-19 infection on campus (University of Heidelberg) in 2020/2021. To the right, the app shows a 96-well plate layout colored either by content type (sample, empty, positive or negative control) or by the assigned result. To the left, it shows the results of three tests and one control for each sample. Accumulation of the LAMP product is indicated by the change of color from red to yellow and is measured as a difference in absorbance on two wave lengths. This difference is plotted as a function of time. Besides exploration (highlighting the corresponding lines for each sample), the app allows to manually reassign status, store results, and send them to the server, where they can be queried by the test subjects. The app is provided as an R script; the code and some example data are available on GitHub at https://github.com/anders-biostat/lamp_plate_analysis

Figure 8 shows a screenshot of an example of a more complex LinkedCharts app, which was developed as part of an effort to establish LAMP-based testing for SARS-CoV-2 at Heidelberg University campus [32]. In this project, a colorimetric assay based on loop-mediated DNA amplification (LAMP, [35]) was carried out on microtiter plates. The app allowed lab technicians to inspect the measured change in pH as function of incubation time, to link curves to wells and to patients, to compare replicates, and to check and, if needed, amend automatic result calling (see [33] for details). Such continuous quality control is vital for reliable medical diagnostics and has to be offered in an easy-to-use manner and quick-to-grasp to avoid mistakes from repetition and fatigue.

Here, LinkedCharts turned out to be well suited to quickly develop the app, to continuously refine it while the assay was finalized, and to turn it into a production tool, well integrated into the testing campaign’s databases and result reporting services.

#### LinkedCharts for Open Science

Analyses in computational biology are often complex and involved, making them difficult to explain and even more so to verify. It is not uncommon that neither the peer reviewers nor the readers of a publication are effectively able to double-check a result unless they would be willing to redo the whole analysis themselves. The importance of making all raw data and code available to do that has been often stressed [36], but even verifying a complex analysis is a demanding task.

Published interactive apps for data exploration are hence the next step towards open science. Traditionally, publications illustrate the characteristics of typical data by showing “typical examples”―but whether an example can be considered typical can be quite controversial. A LinkedCharts app in a paper’s online supplement allows readers to chose their own examples rather than relying on the authors potentially “cherry-picked” ones. A second, less obvious, advantage is that interactivity can help clarify the details of a complex analysis.

While we consider the main area of application for the LinkedCharts to be a part of data analysis, it can be used for result presentation as well. For instance, a LinkedCharts app was used as online supplement to the paper of Wang et al. [31], a big-data study aiming at elucidating to which extent evolution of expression regulation acts on transcription and to which extent on translation. Using RNA-Seq and ribosomal footprinting data from three organs, taken from animals of six species, changes in transcript abundance and in translation of transcripts into proteins were quantified and compared. A core idea of the analysis was that the evolutionary changes to transcription and translation may either compensate for each other (thus compensating deleterious changes in one layer by an opposite change in the other), or reinforce each other (in case of adaptive changes). To this extent, a score denoted as $\Delta$ was calculated, which is negative if the between-species difference is lower in the ribosome footprinting data than in the transcriptional data (thus indicating that transcriptional difference are at least partially compensated on the translational layer) and positive if the variance at the ribosomal layer is higher (indicating reinforcement).

The definition of this $\Delta$-score is technical, and it is hard for the reader to form an intuition on its meaning. By “playing around” a bit with the app, available at https://ex2plorer.kaessmannlab.org/ (static picture: Fig. 7), this is quickly remedied: the reader can click on any gene in the upper scatter plot, inspecting examples of genes with positive, negative, or near-zero $\Delta$-score to see the data from the individual samples. After a few clicks, the relationship between the transcriptional and the translational data on the one hand and the $\Delta$ score on the other hand will be clearer than after reading several paragraphs of text. The use of HTML design elements to position explanatory labels renders the app nearly self-explanatory. Here, it is not a simple picture, but an interactive one, that is worth the proverbial thousand word.

LinkedCharts apps can be used as paper supplements in two ways. As it was described previously, any R/LinkedCharts app supports concurrent use and therefore can be made available for public usage with a very few changes to the code. Alternatively, a user with the knowledge of JavaScript can use the *linked-charts.js* library which is a foundation of R/LinkedCharts to make an app fully contained within an HTML file, as the aforementioned supplement to Wang et al. [31]. Though this approach requires considerably more effort, the resulting app is extremely easy to share, does not require any form of installation, and can be run in any modern web browser. The interface of *linked-charts.js* in many aspects is the same as of R/LinkedCharts, which facilitates the code transformation. To give readers a feeling of similarity between R code of R/LinkedCharts and JavaScript apps of *linked-charts.js*, for every example in the supplement, we provided code for the both languages.

## Summary and conclusion

The importance of using interactivity in data exploration has been discussed since long. In bioinformatics, applications to perform specific analyses for specific data types often offer useful interactive features for data exploration. However, wherever fitting special-purpose tools are not available, analyses are still conducted using static plots, and general-purpose frameworks for interactive visualization are, if at all, only used for presentation of the details of an already finished analysis. The reason that general-purpose frameworks for interactive data visualization are rarely used in the actual analysis is twofold: for technical reasons, the most versatile tools are only available for JavaScript, while bioinformaticians typically work with R and JavaScript. Available tools for R miss a crucial feature: linking.

We have presented R/LinkedCharts, a general-purpose framework for interactive data visualization for R that pulls all event handling from the JavaScript core to the R-based development side. This enables bioinformaticians to code arbitrary reactions to user interactions with individual data points in a chart and to thus link several charts. We show that this allows to easily set up apps where an “overview chart” shows the main results and a click on any item in this overview displays details on this element in a “detail chart.” We have discussed numerous ways how variations on this general idea enable powerful data analysis strategies that can be easily incorporated into a data analyst’s existing work routine. We have argued that the consequent use of such techniques allows for improvements at all stages of a project.

## Methods

### Implementation

The JavaScript foundation of R/LinkedCharts is built on top of the D3 library [10].

*linked-charts.js* is by itself a fully functional tool for interactive data visualization that can be used by those familiar with JavaScript to create stand-alone apps. The library is open-source and available on GitHub at https://github.com/anders-biostat/linked-charts.

The “jrc” package package is used as a bridge between R and JavaScript. It allows one to run JavaScript code from an R session and vice versa. It also manages client connections to the app and is responsible for all the functionality necessary to make an R/LinkedCharts app public. “jrc” in turn is based mainly on “httpuv” [39] package to run a local server and ensure a WebSocket connection [40]. A current version can be found at https://github.com/anders-biostat/jrc, an archived one at [41].

R/LinkedCharts (“rlc” package) is an R [42] interface to the JavaScript version of LinkedCharts. In addition to providing access to *linked-charts.js* functionality, it also ensures proper storing of charts and serving them to each connected client by extending “App” class of the “jrc” package. “rlc” is open source and is available on CRAN or GitHub https://github.com/anders-biostat/rlc.

## Supplementary information

**Additional file 1.** Zip file containing the interactive supplement.

**Additional file 2.** Review history.

## Data Availability

R/LinkedCharts is available as an R package from CRAN, the standard archive for R packages (https://cran.r-project.org/), i.e., it can simply be installed with install.packages("rlc"). No further installation is required, as all components, including the web server and the functionality to link to the web browser, are included in the package and started automatically. For an archived version of the software, see [37]. R/LinkedCharts, as well as its dependency *jrc* and its variant *linkedchart.js* (see the “Methods” section) are open-source software, made availabe under the GNU General Public License version 3 (GPL3). Codes and detailed explanations for all examples discussed in this paper are given in the paper’s interactive supplement, which is also available at https://anders-biostat.github.io/lc-paper/. Several detailed usage tutorials are available at https://anders-biostat.github.io/linked-charts/. The dataset used for example in Fig. 1 is available on the European Read Archive (ERA) under accession PRJEB7455 (secondary accession: ERP007185). The count data have been downloaded from the *recount2* project [38] at https://jhubiostatistics.shinyapps.io/recount/. The dataset for example in Fig. 5 was obtained from the authors. All the data necessary to recreate the example apps are provided in the Supplement of the paper along side the corresponding code. The supplement is available at https://anders-biostat.github.io/lc-paper/ and as Additional file 1 at the Journal’s web site.
