# Supplementary material for: Simple but powerful interactive data analysis in R with R/LinkedCharts
Source: Genome Biol. 2024 Feb 5;25:43. doi: 10.1186/s13059-024-03164-3 (PMC10840235; doi:10.1186/s13059-024-03164-3)
Supplement: Supplementary file 1 — Additional file 1. Zip file containing the interactive supplement. [file 13059_2024_3164_MOESM1_ESM.zip › index.html]

LinkedCharts - Supplement to the paper


# LinkedCharts - Supplement to the paper

This page provides live versions of the figures from the main paper. Each example is given as a *full* version with all the decorations, labels and titles and as a *minimalistic* app with only essential features.

For each example, one can look at the full code requried to generate the app both as an R script and as a JavaScript code. To run the R code, the "rlc" package must be installed from CRAN, or GitHub for the most recent version. The R code can also be run in Jupyter notebooks (with the IRkernel installed); however, since such R sessions are not considered interactive, one must explicitly tell the R session to wait for the messages from the app. To this end, the "rlc" package provides the "listen" function. The JavaScript code relies on the *linked-charts.js* library that can be downloaded from here. Links to all the required data for each example are given in the description below.
