# Supplementary material for: Simple but powerful interactive data analysis in R with R/LinkedCharts
Source: Genome Biol. 2024 Feb 5;25:43. doi: 10.1186/s13059-024-03164-3 (PMC10840235; doi:10.1186/s13059-024-03164-3)
Supplement: Supplementary file 1 — Additional file 1. Zip file containing the interactive supplement. [file 13059_2024_3164_MOESM1_ESM.zip › examples/basic_synt/JS_code_full.html]

```
lc.scatter()
   .x(i => +iris[i].sepal_length)
   .y(i => +iris[i].petal_length)
   .size(i => +iris[i].sepal_width * 2)
   .colourValue(i => +iris[i].petal_width)
   .symbolValue(i => iris[i].species)
   .place();
```
