# Supplementary material for: Simple but powerful interactive data analysis in R with R/LinkedCharts
Source: Genome Biol. 2024 Feb 5;25:43. doi: 10.1186/s13059-024-03164-3 (PMC10840235; doi:10.1186/s13059-024-03164-3)
Supplement: Supplementary file 1 — Additional file 1. Zip file containing the interactive supplement. [file 13059_2024_3164_MOESM1_ESM.zip › examples/oc_de/R_code_min.html]

```
countsums <- colSums(countMatrix)
openPage(useViewer = FALSE, layout = "table1x2")
selGene <- 1915

lc_scatter(
  x = voomResult$AveExpr,
  y = voomResult$tissuetumour,
  colour = ifelse(voomResult$adj.P.Val < 0.1, "red", "black"),
  on_click = function(i) {
    selGene <<- i
    updateCharts("A2")
  },
  place = "A1"
  )

lc_scatter(dat(
    x = sampleTable$patient,
    y = countMatrix[selGene, ] / countsums * 1e6 + .1,
    logScaleY = 10,
    colourValue = sampleTable$tissue,
    title = rownames(countMatrix)[selGene]),
  place = "A2")
```
