# Supplementary material for: Simple but powerful interactive data analysis in R with R/LinkedCharts
Source: Genome Biol. 2024 Feb 5;25:43. doi: 10.1186/s13059-024-03164-3 (PMC10840235; doi:10.1186/s13059-024-03164-3)
Supplement: Supplementary file 2 — Additional file 2. Review history. [file 13059_2024_3164_MOESM2_ESM.docx]

**1^st^ round**

**Reviewer 1**

The paper by Ovchinnikova et al. nicely articulates the need for interactive visualizations. However the concept of linked charts has been around for some time. They can be generated using Vega-Lite (and also Altair in Python), Bokeh, plotly and other libraries. In this manuscript authors describe am R wrapper for an actual JavaScript library LinkedCharts, which is in turn based on D3 framework. I think that while both products (the library itself and its R implementation) are very useful, the paper provides no discussion of the existing solutions that directly compete with this tool. Specifically I am talking about observableHQ (developed by the same person, Michael Bostock, who developed D3) notebook environment. Can authors create an observable exampel with their data? In such an example, would LinkedCharts be necessary, or D3 (or perhaps simpler Vega-Light schema) would be sufficient? The key in making interactive visualization usable by end users, is to minimize setup requirements. With observable such requirements are minimal (as is with JupyterLite). Thus, while I appreciate the amount of work that has been put in into creation of the library, I am a bit less clear on selling point from the stand point of the end user. My specific requests are to provide links from the paper to:

- Incorporate discussion on ObservableHQ and serverless notebooks
- An observableHq demo of LinkedCharts
- A ready to use Jupyter (R kernel) notebook, that implements rlc examples

**Reviewer 2**

In this manuscript, Ovchinnikova and Anders describe R/LinkedCharts, a new software tool to enable exploration of large-scale datasets by "linking" multiple graphical representations of a dataset that can be easily mined to find patterns, explore particular data features, assess data quality, or identify new hypotheses. The authors present multiple examples of how R/LinkedCharts could be deployed on a variety of datasets, ranging from single-cell RNAseq to drug screening experiments - thus demonstrating the flexibility of the software package and also illustrating its utility to a broad audience. The associated resources and software provided with the manuscript are comprehensive and well annotated, including github repo and R cran package. Given the explosion of various high-throughput, multidimensional datasets in the biomedical community, this sort of software tool is likely to be of high-interest to both computational and experimental biologists. Below are a minor issues that should be addressed prior to publication.

1. In general, the manuscript is wordy and longer than necessary, particularly given the very nice interactive examples included in the supplement - given the nature of the software tool, these are much more powerful than static images
2. Github.io repo has additional examples not included in the manuscript that are likely to be of high interest, eg: scRNAseq of cord blood samples tSNE plot with radio buttons to highlight different markers. I suggest including this example in the paper because an approach such as this can greatly aid researchers working to identify different cell types and states using canonical biomarkers.
3. Some figures are low resolution, eg Figure 2.
4. The provided supplement should include interactive plots for all examples presented in the figures. Specifically, I was unable to find interactive tools for Figures 6 and 7. These should be made available with the manuscript.
5. Paragraph on p.10, line 52 should be moved to discussion.

**Author’s response**

We would like to thank both Reviewers for their comments on our work.

We were very happy that Reviewer 2 commented very positively about our work. Unfortunately, Reviewer 1 was so far unconvinced that our work offers functionality that is not already available. We hope that with this revision we can convince Editor and Reviewer that LinkedCharts fills an urgent need that so far has been unmet. To so, we will need to discuss some background, and therefore wish to apologize to Reviewers and Editor that these explanations might require some perhaps lengthy detours.

The short version is: Our initial version seems to have failed to make clear to Reviewer 1, what the "unique selling point" of R/LinkedChart is -- namely, to make linking of charts available to bioinformaticians working with R. We have now thoroughly reworked especially the introductory text to clarify why this was not possible so far and why filling this gap is of great value.

For a more extensive discussion, we will go through Reviewer 1's comments in detail.

Reviewer #1 wrote:

“The paper by Ovchinnikova et al. nicely articulates the need for interactive visualizations. However the concept of linked charts has been around for some time.”

Our aim was to not only articulate the need for interactive visualization in general but for linked charts specifically. We agree that the introduction has not made this clear enough, and we have therefore changed it accordingly.

Before continuing, we would like to reiterate here the distinction between the general concept of interactive visualization and the specific feature of linking of charts.

Interactive plots are common-place and are indeed offered by many plotting libraries. In many of them, it is possible for the user to change the appearance of a plot, zoom in, change axis scaling, highlight or hide parts of the data, or the like. Many libraries also provide some linking functionality by offering a limited set of *hard-wired* links, e.g. functionality to link axes, such that zooming in one chart causes the same zoom elsewhere. Some tools even offer brushing, i.e., the functionality that selecting data points in one chart highlights points representing the same data items in all other charts. Of course, such linking has been discussed since long, and implemented, e.g., by Buja et al. (1991) (who then went on to develop the seminal xgobi/ggobi tool).

What we mean by “linked charts” goes an important step beyond that: with R/LinkedCharts, a developer can link two charts such that the user’s interaction with one chart can cause *arbitrary* changes to another chart. Technically, this requires that the framework reports the user’s interaction back to the application’s code so that the developer can implement an event handler that then, e.g., might rerun calculations with changed data and cause several other charts to change.

With this in mind, we would like to now address the Reviewer’s core question, namely whether such functionality is already provided by other plotting libraries.

“They can be generated using Vega-Lite (and also Altair in Python), Bokeh, plotly and other libraries. In this manuscript authors describe am R wrapper for an actual JavaScript library LinkedCharts, which is in turn based on D3 framework. I think that while both products (the library itself and its R implementation) are very useful, the paper provides no discussion of the existing solutions that directly compete with this tool. Specifically I am talking about observableHQ (developed by the same person, Michael Bostock, who developed D3) notebook environment.

Here, we hasten to point out that we fully share the Reviewer's admiration for Mike Bostock’s work. His D3 framework is, after all, the basis of our library. Furthermore, the core novelty of D3, when it came out, was its focus on offering detailed event handling. D3 made it easy to write event handlers that trigger arbitrary changes to any chart within the application as a reaction to arbitrary user interactions, and it provided functionality to not only detect which plot element the user has interacted with but also to link this plot element back to a specific data element.

In this sense, D3 was one of the first versatile frameworks that made possible implementation of chart linking in our general sense.

So, we do understand why Reviewer 1, in essence, asks what needs our R/LinkedCharts framework can fulfill that is not already addressed by Mike Bostock's D3/Observable framework.

Luckily, this question has a very straight-forward answer: Our R/LinkedCharts framework is meant for bioinformaticians working with R, while Bostock's D3 and his Observable platform are designed for programmers working with JavaScript.

In bioinformatics practice, R and Python are the de-facto standard choices as analysis platforms. While considerable debate within the bioinformatics community might surround the choice between R and Python, JavaScript is definitely not widely used in bioinformatical data analysis (and if so, then only for result dissemination, not for analysis).

So, our starting point is a large community of bioinformatics practitioners who are used to working with R as their platform of choice for data analysis, and it is for them that we want to offer a flexible and easy solution to incorporate interactivity and chart-linking into their routine work.

Practitioners will already have written many R scripts to perform specific aspects of an analysis pipeline, and will want to explore their data interactively by selecting data and then triggering a rerun of this analysis code for the selected data. In our paper, we provide many examples where we highlight how already existing R code can quickly be turned into an R/LinkedCharts app.

If bioinformaticians would want to achieve the same with D3, they would have to port their analysis scripts from R to JavaScript, because D3 event handlers have to be written in JavaScript. This is not only cumbersome and time consuming, but also far easier said than done: All the vast power of existing bioinformatics packages for R (the whole of Bioconductor and much more) can no longer be used, and essential standard analysis functionality is simply not accessible from JavaScript.

Of course, the whole point of ObservableHQ (Bostock's new development platform for working with D3 and other tools) seems to be to establish JavaScript as an alternative platform for data analysis; and from a visualization standpoint, its footing on D3 gives it a head start over R. For the actual data analysis, however, there is a reason that most practitioners stick to R, and do not consider JavaScript a viable alternative.

Granted: Debates on which programming language is "best" for a given purpose are as old as all software engineering, and we might risk starting another one here. Hence, we simply point out that, for better or worse, biology as a field has long settled on R (and Python), and as a JavaScript library, D3, despite its power, is of little use for bioinformatics. Our work changes this: by wrapping around D3, R/LinkedCharts now makes the power of D3-like charts available to bioinformatics in a manner that allows seamless integration into existing workflows.

We feel that this is of tremendous use for bioinformaticians.

We assume that the Reviewer is one of the rare bioinformaticians who know how to do data science in JavaScript and even enjoy that, and hence overlooked the value of being allowed to stay with one’s familiar R environment. We therefore ask the Reviewer to remember that most bioinformaticians are not experienced with JavaScript and, if at all, use it only if it cannot be avoided, i.e., if the script has to run in a browser.

Thinking about the Reviewer's comments made us realize that all the points just made are far from obvious for a reader coming from the "JavaScript world" and apologize for having neglected this perspective. We have revised our introduction and hope that we now make this point much clearer. Also, we have added a new section "Event handling in R" that explains why all available tools that could be used to make linked charts so far require the developer to work in JavaScript, and why it was non-trivial to pull this functionality to R.

In closing this point, we would like to comment on the various other frameworks that the Reviewer mentioned. We went through all of them to check whether some of them might indeed have "scooped" us, but found that the limitations just discussed apply to all of them:

The Reviewer mentions the Python library, Bokeh, which does allow specification of event handlers – but these have to be written in JavaScript, or selected from a short list of hard-wired options. Hence, if one tried to implement chart linking with Bokeh, one would not only have to switch language from Python to JavaScript in order to specify custom event handling but also to manually implement data transfer between two VMs. The very same is true for the other widely used interactive plotting framework for Python, Plotly. We noticed that the developers of these frameworks have also started very recently to work on functionality towards offering full customizable event handling within the respective native environment, but these options do not yet seem to be mature. Finally, the Reviewer mentioned Vega: its descriptive domain-specific language can support only very limited calculations. Porting complex data analysis procedures from R to Vega might be even less possible than porting them to JS.

We now come back to the Reviewer’s request:

“Specifically I am talking about observableHQ (developed by the same person, Michael Bostock, who developed D3) notebook environment. Can authors create an observable exampel with their data? In such an example, would LinkedCharts be necessary, or D3 (or perhaps simpler Vega-Light schema) would be sufficient?”

We hope that with the above, we were able to convince the Reviewer that D3 alone would not be sufficient -- at least not for an analyst used to working in R.

Of course, D3 is necessary, as it is a heart-piece of R/LinkedCharts -- and for a data analyst who has chosen to fully work in JavaScript, it would be the tool of choice. But for our target audience, bioinformaticians working with R, it is R/LinkedChart's connecting D3 to R that makes it useful.

We agree that the simplified examples used in our initial examples could as well have been written in JavaScript as in R, and hence could be put into an ObservableHQ notebook – but this would not at all be convenient, if specific analysis routines have been written in R, which is the case more often than not in bioinformatics practice.

The Reviewer raises an important issue in continuing:

“The key in making interactive visualization usable by end users, is to minimize setup requirements. With observable such requirements are minimal (as is with JupyterLite). Thus, while I appreciate the amount of work that has been put in into creation of the library, I am a bit less clear on selling point from the stand point of the end user.”

For a bioinformatician working in R, the installation of R/LinkedCharts is as simple as could be. R/LinkedChart is provided as a package on CRAN, the standard repository for R packages, and can be installed like any other R package, simply by typing *install.packages(”rlc”)*.

This pulls the package, including all JavaScript parts. The framework runs its own web server within the R process; i.e., no installation of a web service is needed as long as R/LinkedCharts is used locally.

If the interactive visualization should be made available over the WWW, this can also simply be done by running a standard R process on a server and opening a port.

We have added a sentence to the manuscript to make this clear.

We hope that these remarks set the groundwork to discuss the Reviewer’s specific requests: “My specific requests are to provide links from the paper to:

- Incorporate discussion on ObservableHQ and serverless notebooks

As much as we see the convenience of serverless notebooks, they are an option only to developers working in a browser-based language, i.e., JavaScript, and as this is not our target audience.

In fact, the JavaScript core of LinkedChart does allow to produce stand-alone (serverless) pages, and the paper's interactive supplement has, in fact, been produced that way. However, we have now removed most of the discussion of this possibility from the revised paper and now only mention it in passing, because the Reviewer has convinced us that we should concentrate on our target audience, analysts working with R, because JavaScript developers would probably be better served with, e.g., ObservablePlot.

In the Introduction, we now mention Observable Plot as one of the possible solutions for interactive visualization with javaScript.

“- An observableHq demo of LinkedCharts”

We might be able to show how the “inner” JavaScript API of LinkedCharts could be used within a JavaScript-based notebook such as ObservableHQ, and we might have tried to do so two years ago. However, with ObservablePlot now being available, the Reviewer’s own arguments show why there is little value in this. We wish to present R/LinkedChart to the community of R-using bioinformaticians. The Reviewer is very right that data scientists not working with R but with JavaScript are better served by ObservablePlot or perhaps Plotly than by our non-R based “inner” LinkedCharts.

Therefore, we hope that the Reviewer agrees with our decision to focus on using R/LinkedCharts used in development environments for R (e.g., RStudio, Jupyter) and not in those meant only for JavaScript (ObservableHQ).

“- A ready to use Jupyter (R kernel) notebook, that implements rlc examples”

We thank the reviewer for this request as it made us notice a subtle issue with JupyterLab that made it impossible to run the examples shown on the web page also on JupyterLab. We have now added an explanation in the supplement and on our GitHub page on how to do so.

However, we have to concede that even from JupyterLab, the visualization will pop up in a dedicated browser window rather than in a notebook cell. This arguably looks nicer in RStudio where they can be made to appear in RStudio's "Viewer" pane.

*To summerize:* The Reviewer’s comments showed us that we were not clear enough in defining our target audience. The target audience is bioinformaticians working with R, not web developers working with JavaScript. The latter are certainly better served with D3/Observable. We have thoroughly edited the text to make this now clear.

We have also changed the title. It now reads: "Simple but powerful interactive data analysis in R with R/LinekdCharts"

Reviewer 2

We thank Reviewer 2 for their praise for our work:

“Reviewer #2: In this manuscript, Ovchinnikova and Anders describe R/LinkedCharts, a new software tool to enable exploration of large-scale datasets by "linking" multiple graphical representations of a dataset that can be easily mined to find patterns, explore particular data features, assess data quality, or identify new hypotheses. The authors present multiple examples of how R/LinkedCharts could be deployed on a variety of datasets, ranging from single-cell RNAseq to drug screening experiments - thus demonstrating the flexibility of the software package and also illustrating its utility to a broad audience. The associated resources and software provided with the manuscript are comprehensive and well annotated, including github repo and R cran package. Given the explosion of various high-throughput, multidimensional datasets in the biomedical community, this sort of software tool is likely to be of high-interest to both computational and experimental biologists.”

We are very happy to see that the Reviewer sees so clear potential and value in our work. We are also happy that she/he only sees a few “minor” issues:

“Below are a minor issues that should be addressed prior to publication.

1. In general, the manuscript is wordy and longer than necessary, particularly given the very nice interactive examples included in the supplement - given the nature of the software tool, these are much more powerful than static images”

We apologize for our verbosity, and thank the Reviewer for the nice words on our supplement. We agree: our interactive examples are a better advertisement of our tool than a long text, and for explaining the usage, an HTML tutorial is a better medium than a PDF text.

However, an article in a scholarly journal serves two purposes. First, to advertise the work, but also, to justify and defend it. Hence, we felt the need to explain our design choices, discuss the work’s value within the practice of academic bioinformatics, and compare with other approaches.

Nevertheless, we agree that the manuscript was too verbose and therefore went through the text and tried to shorten it, hopefully with some success. If necessary, we could further shorten the description of the example use cases. We would like to ask the Editor for guidance here for the journal’s target page count. If they wish, we will certainly try to remove some more material.

“2. Github.io repo has additional examples not included in the manuscript that are likely to be of high interest, eg: scRNAseq of cord blood samples tSNE plot with radio buttons to highlight different markers. I suggest including this example in the paper because an approach such as this can greatly aid researchers working to identify different cell types and states using canonical biomarkers.”

We thank the Reviewer for looking at these examples, too, and for their kind words of praise for them. We did not wish to blow up the paper even more and had hence refrained from adding static figures to the main article to advertise these examples, too. However, the Supplement has space, and we have hence now added a panel to the Supplement with links to these extra examples, and a sentence to the main text, that the web page contains more examples. We hope that this will help the readers discover these examples.

“3. Some figures are low resolution, eg Figure 2.”

Thanks for pointing this out. We increased the resolution of Figure 2, as well as of Figure 1 and Figure 6.

“4. The provided supplement should include interactive plots for all examples presented in the figures. Specifically, I was unable to find interactive tools for Figures 6 and 7. These should be made available with the manuscript.”

Figure 6 refers to an app that is published as a part of a paper and is maintained by its authors. We now include a link to the live version of the app. We now also mention it in the Supplement with a link to a live version and a link to the GitHub repository with the source code. However, this particular app is largely based on JavaScript (not only on LinkedCharts, but also on the D3 library) and therefore we feel that a detailed explanation of its code would be out of the scope of the paper.

The app from Figure 7 cannot be included in the supplement for the opposite reason. It relies on the connection with an R session and cannot be part of a stand-alone supplement. We added the mentions of it to the supplement with a link to the GitHub repo that contains code, example data and instructions on how to run the app.

“5. Paragraph on p.10, line 52 should be moved to discussion.”

Thanks, we agree. In fact, the entire section to which the paragraph belonged is now removed from the paper, and the content is now only briefly mentioned in the Discussion.

**2^nd^ round**

**Reviewer 1**

With their response the authors have convinced me in the validity of their work.
